# The Agony of Choice? Preserved Affective Decision Making in Early Multiple Sclerosis

**DOI:** 10.3389/fneur.2020.00914

**Published:** 2020-09-02

**Authors:** Nils C. Landmeyer, Inga Dzionsko, Laura Brockhoff, Heinz Wiendl, Gregor Domes, Jens Bölte, Julia Krämer, Sven G. Meuth, Andreas Johnen

**Affiliations:** ^1^Department of Neurology With Institute of Translational Neurology, University Hospital Münster, Westfälische-Wilhelms-University Münster, Münster, Germany; ^2^Department of Biological and Clinical Psychology, University of Trier, Trier, Germany; ^3^Department of Psychology, Westfälische-Wilhelms-University Münster, Münster, Germany

**Keywords:** multiple sclerosis, post-error slowing, Iowa gambling task, somatic marker hypothesis, reward processing, cognition

## Abstract

**Background:** Cognitive impairment (CI) is an early and frequent symptom of multiple sclerosis (MS). Likewise, affective symptoms (e.g., depression and anxiety) and alterations in the processing of emotional stimuli have been frequently reported. Thus, abilities that integrate affective and cognitive processes such as decision making (DM) based on affective feedback are potentially valuable early diagnostic markers for MS. The available research on this topic, however, is still inconclusive and suffers from methodological issues.

**Methods:** We compared DM ability in a clinically homogeneous cohort of 24 patients with early relapsing–remitting MS (RRMS) and 59 age-matched healthy controls (HCs). A modified version of the Iowa gambling task (IGT) allowed us to control for individual differences in search strategies during the risk exploration phase. Besides standard IGT measures (netscore, obtained play money, and learning index), we also examined reaction times and post-error slowing (PES) patterns as a proxy for abnormalities in the processing of affective feedback.

**Results:** The performance of patients did not significantly deviate from HCs in any standard parameter of the modified IGT. Furthermore, although RRMS patients reacted significantly slower than HCs overall, we found similar patterns of PES in both groups, suggesting similarly efficient processing of affective feedback.

**Conclusion:** We conclude that there is no specific deficit in affective feedback processing in early RRMS. Previous findings of IGT impairments in this patient group may thus not represent a genuine deficit in affective DM but rather be related to sample characteristics, general CI, and/or differences in individual search strategies. Future research should explore the potential influence of lesion volumes and locations on DM ability by employing brain imaging techniques.

## Introduction

Multiple sclerosis (MS) is a chronic inflammatory and neurodegenerative disease of the central nervous system involving progressive neurological symptoms like disturbed vision, paresthesia, as well as autonomic and motor dysfunction (1). It is further characterized by a range of cognitive, behavioral, and psychological symptoms that may be present already in early disease stages: In the first 2 years after disease onset, up to 45% of patients exhibit cognitive changes, most notably in the domains processing speed, complex attention, and (working) memory, rendering cognitive impairment (CI) a key symptom of early MS (2). CI has been shown to have adverse effects on the occupational, private, and social contexts of patients (3). Other frequently reported symptoms include depression, anxiety, and fatigue, which may also interact with cognitive performance (4). These affective and emotional changes represent another core problem of early MS (5) and have been related to alterations in the perception and processing of emotional stimuli (6). As a result, interpersonal functioning and the psychological and social aspects of quality of life can be affected (7).

Abilities that integrate cognitive and affective processes like decision making (DM) may thus be particularly vulnerable in early MS (8). DM has been defined as the ability that allows persons to form preferences, to select and execute actions, and to evaluate the affective outcome of a selected choice (9). This ability relies on both cognitive functions (e.g., attention, executive functions, and memory) and on the perception and processing of affective feedback alike.

A framework about how affective feedback and cognitive function interact is offered by the somatic marker hypothesis (SMH) (10). The SMH postulates that the outcomes of decisions are unconsciously translated into changes in bodily states (e.g., heart rate), indicating either a positive or a negative affective valence. These physiological changes accompany emotional reactions and allow us to swiftly evaluate the incentive value of a decision, which is especially relevant in complex situations in which cognitive resources can be overstrained. Therefore, somatic markers are able to enhance the accuracy and efficiency of human decision making by preselecting favorable outcomes and guiding decisions away from disadvantageous choices. Importantly, somatic markers develop through previous encounters with similar situations (11). An efficient processing of these somatic markers thus ultimately results in an implicit learning of risk contingencies, leading to advantageous decisions and to the avoidance of unfavorable high-risk decisions.

The physiological underpinnings of affective DM have been associated with a large neural network including the ventromedial prefrontal cortex (VMPFC), the orbitofrontal cortex, as well as the striatum and the amygdala (12). Recent evidence then suggests that the key areas of this network are also early and specific targets of neuroinflammation and neurodegeneration in MS, again emphasizing the potential relevance of assessing DM in this group of patients (13).

The Iowa gambling task (IGT) has been most frequently employed to assess affective DM ability. In this computerized card game, participants are instructed to earn as much play money as possible by drawing cards from one of four different decks. The decks differ in terms of the risks of winning or losing play money of various amounts (14). The underlying risk contingencies are unknown to the participants. They have to be explored and implicitly learned during the course of the task *via* processing of affective feedback (i.e., by winning or losing play money following a decision).

Studies investigating the IGT performance of MS patients, however, draw an inconclusive picture. Some studies did not find differences regarding DM performance when comparing MS patients and healthy controls on the IGT (15, 16). For instance, Simioni et al. observed no differences in IGT performance in a sample of 109 relapsing–remitting MS (RRMS) and 56 patients with clinically isolated syndrome compared to controls. Other studies found undisturbed performances in the early phases of the IGT (i.e., when risk contingencies regarding winning or losing were still unexplored for participants), but impaired performances in the later phases of the task in patients with MS (17, 18). These latter results have been discussed as potentially reflecting an inefficient learning from affective feedback (17). Conversely, other research suggested a general impairment of MS patients on the IGT, i.e., patients chose advantageous decks less frequently compared to healthy controls throughout the task (19–21).

Studies using the original IGT, however, may suffer from methodological shortcomings, hampering a clear-cut interpretation of the results. First, a known and critical issue of the original IGT is that the task does not account for individual differences in search strategies during the exploration of risk contingencies. Patients with MS could thus employ fundamentally different exploration behaviors during the early phases of the task by, e.g., drawing cards from only some of the available four card decks while completely or partially ignoring other decks. As a result, the risk contingencies of all four decks may not be explored efficiently, resulting in unfavorable decisions despite preserved abilities to learn from affective feedback. Such different search strategies have previously been reported in younger children as compared to adults (22). Secondly, previous studies only evaluated the accuracy measures of the IGT (e.g., total earned money) and neglected other measures that could provide a deeper insight into the processing of affective feedback (e.g., reaction times following winning or losing trials). Finally, most available studies on DM ability in MS employed clinically heterogeneous samples with regard to disease severity and disease course (17, 23, 24). This is particularly problematic given that the rates of CI differ remarkably between relapsing-remitting and progressive disease courses, which may also influence DM performance (25).

Here, we assessed DM in a clinically homogeneous RRMS cohort with a disease duration of <2 years. We controlled for potential differences regarding the search strategies and exploration behavior by using a modified, forced-choice version of the IGT (22). We further analyzed the general processing speed ability by using the symbol digits modalities (SDMT) (26) test as well as the specific reaction time patterns on the IGT as a potentially sensitive marker for affective feedback processing ability.

## Materials and Methods

### Participants

Twenty-four consecutive patients (17 females) with early RRMS fulfilling the revised McDonald criteria (27) and 59 (36 females) healthy volunteers underwent neuropsychological examination. RRMS patients were treated at the Department of Neurology with the Institute of Translational Neurology, University Hospital Münster, Germany, and volunteered to participate in cognitive research. To evaluate physical disability, a trained and certified neurologist assessed the Expanded Disability Status Scale (EDSS) scores. All patients exhibited only mild visual and motor involvement that did not interfere with the cognitive assessments. All patients were right-handed and were screened for the following exclusion criteria before inclusion: relapses or systemic therapy with steroids within 1 month before cognitive assessment; history of or current psychiatric disorders (e.g., major depression and substance addiction); and other neurological and medical conditions involving brain pathology.

The HCs were matched for age and consisted of psychology students recruited by bulletins at the University of Trier, as well as employees of the University Hospital of Münster. Neuropsychological assessments were carried out at the University of Trier at the Faculty of Biological and Clinical Psychology and at the University Hospital of Münster at the Department of Neurology. The study was approved by the ethics committee of the University of Münster and the Physicians' Chamber of Westphalia-Lippe (2017-754-f-S). All participants gave written, informed consent.

### Modified Iowa Gambling Task

The IGT is a card selection paradigm to evaluate decision-making processes which has been frequently employed in a variety of neurological populations, including Parkinson's disease, Huntington's disease, and MS (28, 29).

In this study, we evaluated DM ability using a modified version of the IGT as described by Cauffman et al. This version of the IGT was originally developed to control for potential differences regarding search strategies and exploration behaviors as, e.g., younger children tend to play cards repeatedly from the same decks. Similar to the original IGT, participants were instructed to gain as much play money as possible by drawing cards from either of four decks displayed on a computer screen. Card decks C and D were statistically advantageous, resulting in long-term gains, in contrast to decks A and B that led to long-term losses. The exact underlying risk contingencies of the task are depicted in Table 1, but were not disclosed to the participants. To control for the possibility of different search strategies in MS patients, participants could only decide to play or pass on a predetermined deck (indicated by an arrow on the screen), but could not choose freely which card deck they wanted to play (forced choice). Each decision thus resulted in one of four possible outcomes: gaining money after deciding to play, losing money after deciding to play, no deposit change after deciding to play, or no deposit change after passing on a card deck. The current total amount of money and the amount of money earned in the preceding trial were displayed below the decks, on the screen. All participants completed 120 trials in six blocks of 20 cards each.

**Table 1:** Deck characteristics and risk contingencies of the modified Iowa gambling task as reported by Cauffman et al. (22).

	**Deck A**	**Deck B**	**Deck C**	**Deck D**
Payoff range (in €)	−250 to 100	−1,150 to 100	−25 to 50	−200 to 50
Probability of gain	0.50	0.90	0.50	0.90
Probability of loss	0.50	0.10	0.25	0.10
Probability of 0€ payoff	0.00	0.00	0.25	0.00

The main outcomes of the modified IGT were the *netscore* after each block and the *total netscore* (averaged over all six blocks), as well as the *amount of play money* after each block and the *total amount of play money* (after completing all six blocks). The netscore was calculated by subtracting the number of cards selected from the disadvantageous decks (A and B) from the number of cards drawn from the advantageous decks (C and D). It is suggested that the IGT evaluates DM in a 2-fold approach that differentiates between decision making under ambiguity in the first two blocks and decision making under risk in the following blocks. Decision making under ambiguity is characterized by an initial lack of knowledge about the probabilities for specific outcomes as participants follow their guesses and hunches to choose between decks, while decision making under risk considers implicitly known probabilities (30). Thus, the mean netscore performance for DM under *ambiguity* (block 1 and block 2) and under *risk* (block 3, block 4, block 5, and block 6) was also calculated, as some evidence suggests pronounced deficits only in the later phases in RRMS (19). In a similar fashion, a modified learning index ([(B3 + B4 + B5 + B6)/4] – [(B1 + B2)/2]) was calculated to test a potential change in performance during the course of the IGT (16). Finally, the *percentage of played cards* (as opposed to pass trials) in each block was evaluated as another marker for exploration behavior.

### Reaction Times and Post-error Slowing

To evaluate more closely the efficiency of learning from affective feedback and, thus, the cognitive processes underlying affective DM in MS, we also analyzed the reaction times and post-error slowing (PES) patterns. The concept of PES describes an increase in reaction times in trials following an error (here: after a negative affective feedback, i.e., loss of play money) (31). Evidence suggests that PES is closely associated with somatic markers, including a decrease in heart rates (32). Alterations in PES may therefore represent an indicator for an inefficient processing of negative affective feedback, which may consequently result in disadvantageous future decisions.

To evaluate PES patterns within the IGT, the mean reaction times following *post-loss* and *post-gain* trials were calculated (33). Loss trials were operationalized as trials in which choosing a card deck resulted in losing money (i.e., negative affective feedback), whereas *gain* trials were trials in which money was won. To model also the strength of negative affective feedback, trials in which the participants lost up to 150€ were further categorized as *low-loss* and trials in which 150€ or more were lost were summarized as *high-loss* trials, resulting in the three-level factor *trial type* (*post-gain, post-low loss*, and *post-high loss*). Situations in which the participants neither earned nor lost money or passed on a deck were not considered for the PES analysis. Trials with implausible fast reaction times <250 ms and trials in which no decision was made within the time limit of 4,000 ms were *a priori* excluded from this analysis.

### Neuropsychological Assessment

All participants underwent a brief cognitive screening including the written Symbol Digits Modalities Test (SDMT) as a measure of cognitive processing speed. The SDMT is recommended by experts as the most sensitive single screening test for overall cognitive impairment in MS (34). To evaluate patients' working memory abilities, the Paced Auditory Serial Addition Test (PASAT 3s) was additionally employed (35). Performances in the SDMT and PASAT 3s were considered as impaired when the calculated normative *z* score was below −1.645 (representing the fifth percentile rank) (36, 37).

Furthermore, mood characteristics were assessed using the Hospital Anxiety and Depression Scale (HADS), a questionnaire to screen for anxiety disorders and depression particularly in patients with somatic and psychiatric illnesses (38). MS-related cognitive and motor fatigue symptoms were additionally evaluated using the Fatigue Scale for Motor and Cognitive Functions (FSMC) (39).

### Statistical Analysis

All conducted analysis and results can be accessed *via* the Open Science Framework (OSF; https://osf.io/3kcqv/).

The statistical analyses were performed using the RStudio (version 1.2.1335) and SPSS software (version 26) for Mac OS X (40, 41). Normal distribution and homogeneity of variance were assessed using the Kolmogorov–Smirnoff and Levene's tests. Differences regarding the demographic, clinical, and neuropsychological characteristics as well as the performances on the IGT of the RRMS and HC groups were assessed using unpaired *t*-tests and chi-squared tests. To evaluate group differences regarding PES, a mixed 2 × 3 repeated measures ANOVA design with the factors *group* (HC vs. RRMS) and *trial type* (post-gain, post-low loss, and post-high loss) was employed. Furthermore, Bonferroni-corrected *post hoc* comparisons between the post-gain, post-low loss, and post-high loss trials using paired *t*-tests were calculated.

## Results

### Demographic and Clinical Characteristics

The demographic characteristics of the RRMS patients and the HCs are depicted in Table 2. RRMS patients had a mean age of 33.21 years (SD = 10.75) compared to a mean age of 29.85 years (SD = 12.26) in HCs. The time between diagnosis of MS and examination date was 1.89 years (SD = 0.92), on average. Patients had only mild physical disability, indicated by a median EDSS of 1.25 [interquartile range (IQR) = 1, Min = 0, Max = 3.5]. None of the patients exhibited major motor involvement or visual disturbances that interfered with the cognitive assessment. MS patients and HCs significantly differed regarding cognitive processing speed abilities, as assessed by the SDMT [RRMS: *M* = 51.42, SD = 10.24; HC: *M* = 59.67, SD = 10.19, *t*_(80)_ = 3.3, *p* < 0.01]. Evaluation of normative *z* scores revealed that four of the RRMS patients (16.67%) and one of the HCs (1.69%) reached a *z* value below −1.645 (representing the fifth percentile rank), hinting to a clinically significant cognitive impairment in the domain of processing speed. Furthermore, the mean PASAT 3s score of the RRMS patients (not assessed in HCs) was 45.92 (SD = 13.65), with three patients (12.5%) attaining a *z* value below the fifth percentile rank. In addition, patients and HCs slightly differed regarding years of education [RRMS: *M* = 11.65, SD = 1.65; HC: *M* = 12.96, SD = 0.33, *t*_(23.74)_ = 3.9, *p* < 0.01]. No other differences in the clinical or demographic parameters emerged between both groups. Critically, RRMS patients and HCs exhibited comparable and overall low levels of anxiety, depression, and fatigue (Table 2).

**Table 2:** Demographic and clinical characteristics of the RRMS and HC groups.

	**RRMS (*n* = 24)** ***M* (SD)**	**HC (*n* = 59)** ***M* (SD)**	***p*-value**
**Demographics**			
Age (years)	33.21 (10.75)	29.85 (12.26)	0.25
Sex (% female)	70.83	61.02	0.46
Education (years)	11.65 (1.65)	12.96 (0.33)	**<0.01**
EDSS, median (IQR)	1.25 (1)	NA	NA
Disease duration (years)	1.89 (0.92)	NA	NA
Disease-modifying therapies (%)	Fingolimod: 25 Natalizumab: 25 Interferon beta: 16.7 Dimethyl fumarate: 12.5 Glatiramer acetate: 8.3 Alemtuzumab: 4.2 Teriflunomid: 4.2 No treatment: 4.2		
Vocational status (%)	Employed: 83.33 Unemployed: 8.3 Student: 4.2 Unknown: 4.2		
**Neuropsychological assessment**			
SDMT	51.42 (10.24)	59.67 (10.19)	**<0.01**
PASAT 3s	45.92 (13.65)	NA	NA
HADS Depression	3.63 (3.51)	3.39 (3.25)	0.77
HADS Anxiety	5.04 (3.93)	5.79 (3.58)	0.40
FSMC	43.58 (21.31)	43.40 (13.97)	0.97

*RRMS, relapsing–remitting multiple sclerosis; HCs, healthy controls; n, number of patients; M, mean; SD, standard deviation; SDMT, Symbol Digit Modalities Test; PASAT 3s, Paced Auditory Serial Addition Test three-second interval; EDSS, Expanded Disability Scale; HADS, Hospital Anxiety and Depression Scale; FSMC, Fatigue Scale for Motor and Cognitive Functions. IQR, interquartile range. Bold values indicate statistical significance*.

### Standard Accuracy Measures of DM

**Figure 1:**
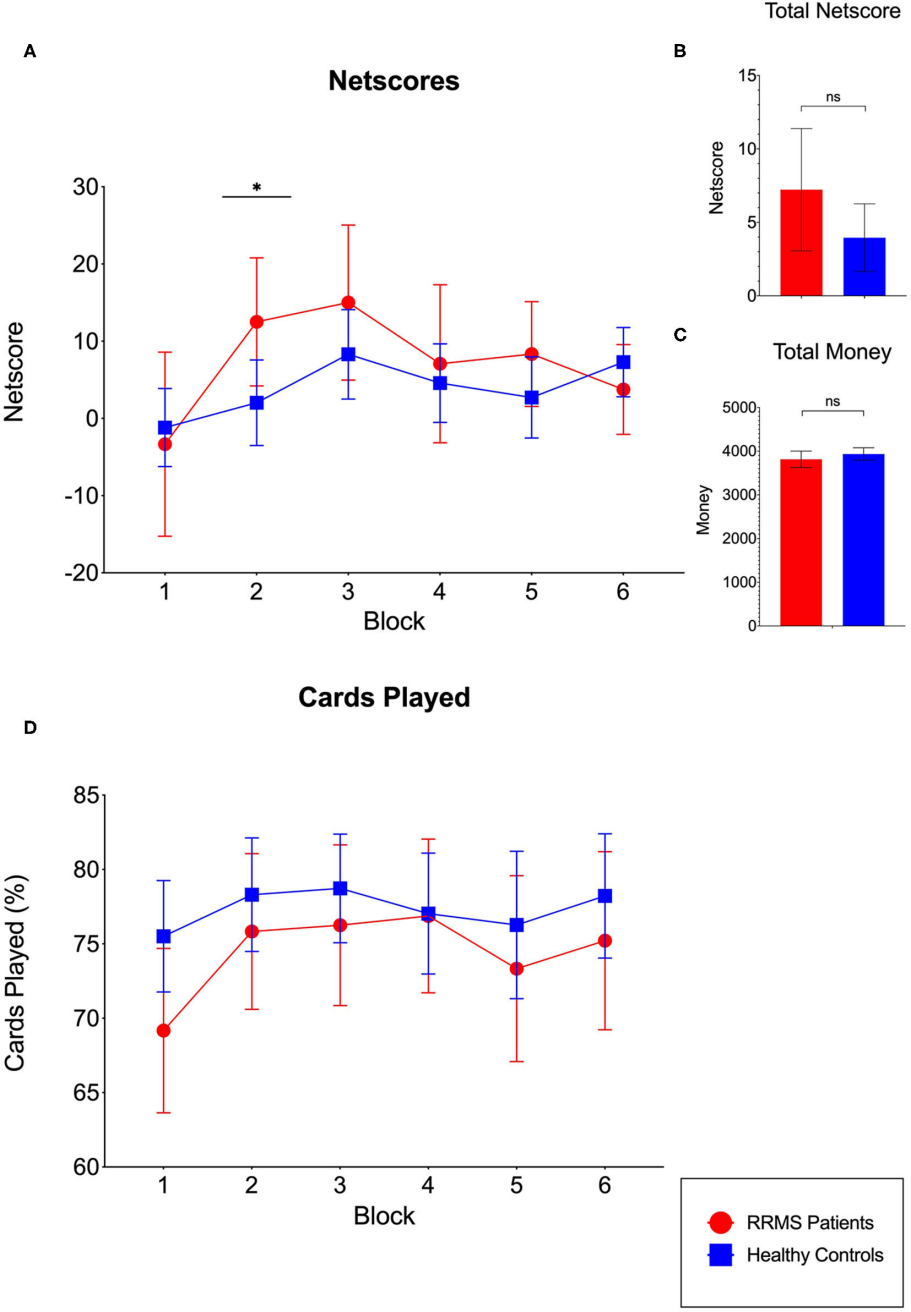
Performance of the relapsing–remitting multiple sclerosis (RRMS) patient group and the healthy control (HC) group in the modified Iowa gambling task (IGT). **(A)** Evolution of the *netscore* (difference between the percentage of advantageous and disadvantageous decisions, with the latter being subtracted from the former) during the six blocks of the task. Only block 2 revealed significant differences, indicating better performance of the RRMS patients. **(B)** The total netscore revealed no significant differences between groups. **(C)** The total amount of money at the end of the task was comparable between the RRMS patients and HCs. **(D)** Evolution of the percentage of cards played during the six blocks of the task. No differences between the RRMS patients and HCs in any of the six blocks emerged.

Both groups showed comparable performances regarding the total netscore [RRMS: *M* = 7.22, SD = 9.85; HC: *M* = 3.95, SD = 8.84, *t*_(81)_ = −1.48, *p* = 0.14] and the total amount of play money [RRMS: *M* = 3,814.58, SD = 442.87; HC: *M* = 3,934.75, SD = 547.09, *t*_(81)_ = 0.96, *p* = 0.34] at the end of the task (Figures 1B,C). To evaluate the evolution of performance during the IGT, the netscores for each of the six blocks were examined (see Figure 1A). Only block 2 revealed a significant difference between the RRMS patients (*M* = 12.50, SD = 19.62) and healthy controls (*M* = 2.03, SD = 21.24), indicating better performance of the RRMS patients in this block [*t*_(81)_ = −2.08, *p* = 0.04].

Furthermore, the comparisons between DM under ambiguity (mean netscore of block 1 and block 2) and under risk (mean netscore of block 3, block 4, block 5, and block 6) revealed no significant differences between groups [ambiguity: RRMS: *M* = 4.58, SD = 16.01, HC: *M* = 0.42, SD = 15.09, *t*_(81)_ = −1.12, *p* = 0.27; risk: RRMS: *M* = 8.54, SD = 10.88, HC: *M* = 5.72, SD = 10.06, *t*_(81)_ = −1.13, *p* = 0.26]. Finally, the modified learning index also revealed no significant differences between groups [RRMS: *M* = 3.96, SD = 17.41, HC: *M* = 5.30, SD = 17.15, *t*_(81)_ = 0.32, *p* = 0.75].

The evaluation of percentage of cards played (Figure 1D) showed a trend of RRMS patients playing fewer cards and, in contrast, pass more often. However, this difference did not reach significance in any of the six blocks.

In addition, Pearson's correlations of the main IGT outcomes (total netscore, total amount of money at the end of the task, and learning index) with the demographic and clinical (age, education, EDSS, disease duration, and HADS) and cognitive outcomes (SDMT and PASAT) revealed no significant relationships (see Supplementary Table 1 for details).

### Evaluation of Reaction Times and Post-error Slowing Patterns

**Figure 2:**
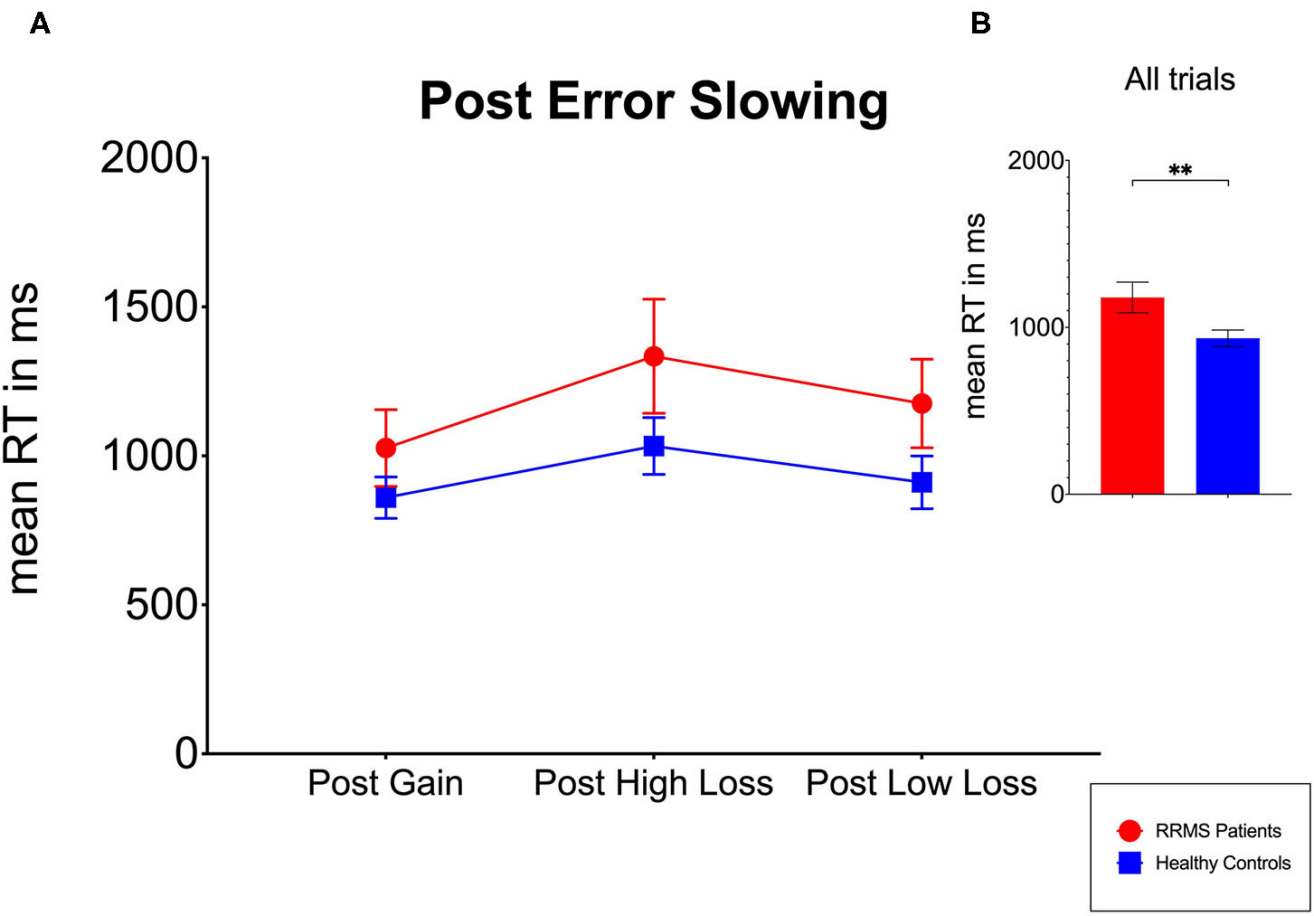
Evaluation of post-error slowing (PES) for RRMS patients and HCs. **(A)** RRMS patients and HCs exhibit a similar pattern of PES as no significant interaction between the factors *group* and *trial type* emerged. **(B)** The overall reaction times in the RRMS patients were significantly higher compared to the HCs. *RT*, reaction times; *RRMS*, relapsing–remitting multiple sclerosis; *HC*, healthy controls. Values are presented as the means. *Error bars* indicate 95% confidence intervals. **p < 0.01.

None of the trials for either patients or HCs were excluded due to exceeding the reaction time limit of 4,000 ms. In the RRMS and HC groups, 0.31% and 2.83% of all trials, respectively, had to be removed due to reaction times faster than 250 ms, which were considered implausible (42, 43). The two-way mixed (2 × 3) ANOVA revealed significant main effects for the factor *group* [RRMS vs. HC: *F*_(1,81)_ = 11.45, *p* < 0.01, η^2^_g_ = 0.10] and for the factor *trial type* [post-gain vs. post-low loss vs. post-high loss: *F*_(2,162)_ = 24.17, *p* < 0.01, η^2^_g_ = 0.07], as displayed in Figure 2A.

For the first main effect (factor group), *post hoc* groupwise comparisons indicated that the RRMS patients (*M* = 1,179 ms, SD = 391) showed overall slower reaction times compared to HCs (*M* = 935 ms, SD = 332) (Figure 2B).

For the second main effect (factor trial type), the Bonferroni-corrected *post hoc t*-tests revealed that the overall reaction times after the post-high loss trials (*M* = 1,120 ms, SD = 413) were significantly higher compared to post-gain (*M* = 908 ms, SD = 286) trials [*t*_(82)_ = −6.05, *p* < 0.01]. Additionally, significant differences emerged between the post-gain and post-low loss (*M* = 988 ms, SD = 361) trials [*t*_(82)_ = −3.11, *p* < 0.01] and between the post-high loss and post-low loss trials [*t*_(82)_ = 3.94, *p* < 0.01].

Critically, however, no significant interaction between the factors group and trial type emerged, pointing to no differences regarding the PES patterns between the RRMS patients and HCs.

## Discussion

The ability to make profound and efficient decisions in personal and occupational contexts is a vital competence. Problems in this domain may be related to both cognitive and affective dysfunctions of patients with MS in the early stages (44). Given that both cognitive and affective processes are crucially involved in this ability, deficits in DM could serve as an early marker for MS. However, previous studies revealed conflicting evidence on whether patients with early MS exhibit a genuine impairment in affective DM abilities. Here, by focusing on an early RRMS cohort with overall low physical disability and motor impairments as well as by employing a modified version of the well-known IGT and an in-depth analysis of the reaction time patterns that served as a proxy for the processing of affective feedback, we aimed to gain greater insight into the underlying processes of DM in this patient group.

The major finding of our study is that patients with early RRMS performed similar to age-matched healthy controls regarding DM on a modified version of the IGT that controlled for individual differences in search strategies and exploration behavior. The total *netscore* (representing the difference between advantageous and disadvantageous decisions averaged over all six blocks) and the *total amount of play money* at the end of the task were comparable in patients and HCs. Similarly, measures of decision making under ambiguity, decision making under risk, as well as a modified learning index revealed no significant differences between patients and HCs. Furthermore, an in-depth analysis of the reaction time patterns revealed no significant differences between patients with MS and HCs regarding the processing of affective feedback. Although MS patients showed slower reaction times overall, both groups exhibited a similar increase in reaction times after negative affective feedback (i.e., after a loss of money following unfavorable decisions). We interpret these results as reflecting efficient learning from affective feedback signals that led to similarly advantageous decisions in patients with early RRMS and HCs.

Our results are in line with previous studies that also did not find deficits in the IGT when examining DM ability in mildly impaired early RRMS samples, indicating preserved DM ability at least in the early disease stages (15, 16). However, our results also contradict studies reporting deficits in DM abilities in MS using the original IGT. Some of these studies included mixed and clinically heterogeneous samples consisting of diverse clinical phenotypes and also progressive forms of MS (17, 23, 24, 45). These patient samples for example had substantially higher mean ages as well as higher levels of physical and cognitive disability compared to our study cohort, rendering the deficits in DM as a potential accessory symptom of a more pronounced global cognitive impairment. However, even in more similar cohorts involving only early RRMS patients with low disability statuses, deficits in DM abilities have been reported (18–20). One potential explanation for this discrepancy to our results is that potential differences regarding the search strategies of patients were neglected in previous studies that used the original IGT. During the first blocks of the task (decisions under ambiguity), patients with MS may employ a more conservative approach to risk exploration and may, thus, not explore all card decks equally but rather choose to play cards from only a limited number of decks. In this scenario, patients may not learn the contingencies of all decks equally well and then ground their decisions on “incomplete data,” resulting in disadvantageous performances, although their DM ability (defined as an efficient learning from affective feedback) may actually be preserved. This hypothesis is supported by studies that observed a pronounced performance deficit particularly in the later stages (decisions under risk) as compared to the initial phase of the IGT, in which risk contingencies were still mostly unexplored for all participants (17, 18, 20). The idea of a more cautious explorative behavior and the avoidance of unnecessary risks of MS patients is supported by a study of Simioni et al. (46) that found a higher risk aversion concomitant with alteration in DM ability, presumably reflecting a reduced faith in MS patients' own choices. In our study, we controlled for such gross differences in search strategies by employing a modified (forced-choice) version of the IGT, in which participants could only decide to play or pass on a preselected deck. Thus, all participants were “forced” to explore the contingencies of all decks equally and in a more comparable fashion.

Apart from abnormal search strategies, other factors could also influence and explain the previously reported deficits in affective DM ability in early RRMS patients (47). Available evidence hints at working memory capacity being a potentially crucial determinant of IGT performance. This idea was substantiated by the finding that deficits in the IGT were more pronounced in patients with damage to the VMPFC with concomitant working memory impairments (48). Interestingly, studies that observed deficits in the IGT in early RRMS patients also reported severe deficits in working memory (18–20). In line with this, our early RRMS sample was characterized by a low prevalence of both processing speed impairment assessed by the SDMT (16%) as well as working memory disturbances measured by the PASAT (12.5%). Other mechanisms that could explain the impaired performance on the IGT in early RRMS are affective symptoms that were often not systematically considered or reported in previous research (18, 20). To take this into account, we tested and found similar levels of anxiety and depression compared to healthy controls in our sample. Studies in both clinical and healthy samples using the IGT finally hint to an association between decision making and alexithymia (49–52). Research suggests a high prevalence of alexithymia in MS patients, implying disturbances in identifying, understanding, and describing their emotional states. As a result, the ability to express emotional needs may be limited (6, 53).

Although we evaluated PES patterns as a proxy for abnormalities in the processing of affective feedback, a potential limitation of our study is that direct physiological measures of affective changes (e.g., heartbeat, skin conductance, and event-related potentials) were not recorded. Therefore, we cannot fully rule out abnormalities in physiological responses or perception of physiological changes within the process of DM in early MS. The inclusion of a direct measure of the emotional component of decision making in combination with IGT outcomes would have allowed a further interpretation of the results in the context of the SMH. To our knowledge, only two studies evaluated skin conductance reactivity (SCR) in combination with measures of DM in MS, with rather conflicting results. One article reported a reduced skin conductance reactivity in DM under ambiguity, while the other observed preserved SCR in DM under risk (17, 46).

Furthermore, due to the lack of brain neuroimaging data in this study, we are not able to draw conclusions regarding the neural underpinnings of decision making in RRMS. Available evidence suggests the frontal lobes, prefrontal lobes, insula, the caudate nuclei, and cingulate gyrus activity are related to the process of decision making (8, 54, 55). Individual differences in lesion volume and locations might also account for some of the observed heterogeneity between studies. This assumption is supported by evidence of impaired decision making in patients with high lesion burden whereas patients with low lesion burden were unimpaired when compared with healthy controls (55). In the same regard, correlations between decision-making outcomes and temporal lesions as well as white matter lesions were observed (23, 54). Also, cognitive reserve could function as an important moderator between the amount of brain damage and the extent of clinical outcomes as it is suggested that greater intellectual enrichment protects against cognitive dysfunction in MS (56).

Finally, it remains debatable altogether whether a computerized card game like the IGT is a valid operationalization of DM considering that DM under real-life conditions appears as unequally more complex and demanding. Previous research furthermore estimated the retest reliability of the IGT in the range between *r* = 0.35 and *r* = 0.65 for a retest interval of 14 days in healthy controls (57). Low retest reliability is a major issue in clinical assessment tools to the extent that they may become virtually useless to reliably evaluate individual performance differences particularly in clinical populations, which may ultimately also result in large between-study variations particularly in studies with small samples (47, 58, 59). The modified version of the IGT used in this study has not been analyzed with regard to reliability, but given that the task controls for individual search strategies by a forced-choice paradigm, we expect a higher retest reliability as compared to a free-choice paradigm.

In summary, our results do not suggest a genuine impairment or alterations of affective DM ability or its underlying cognitive processes in early RRMS. Early RRMS patients thus seem fully capable to process and learn from affective feedback in order to make advantageous future decisions. Preserved affective DM ability in MS may thus be used as an important resource in rehabilitation contexts: Patients are able to evaluate the outcomes of selected choices and are also capable of elaborate decisions regarding, e.g., drug treatments or changes in occupational contexts. To account for potential abnormalities in search strategies and exploration behaviors, patients should, however, be confronted with only a limited number of choices at a time and should be encouraged to explore all potential options equally given that we found evidence of more cautious exploration behavior in patients. For diagnostic purposes, processing speed and working memory appear to be more sensitive and robust early cognitive markers for RRMS than DM abilities. Future studies should evaluate DM ability in distinct subgroups of MS and abstain from investigating mixed samples consisting of both relapsing and progressive phenotypes. To evaluate whether DM difficulties develop parallel to general cognitive dysfunction or independently from it, longitudinal studies including early RRMS and progressive MS cohorts using the modified IGT are needed.

Finally, direct measures of physiological changes following affective feedback in combination with brain imaging techniques should be investigated.

## Data Availability Statement

The datasets generated for this study are available on request to the corresponding author.

## Ethics Statement

The studies involving human participants were reviewed and approved by Ethics committee of the University of Münster and the Physicians' Chamber of Westphalia-Lippe. The patients/participants provided their written informed consent to participate in this study.

## Author Contributions

NL: drafting the manuscript, acquisition of data, statistical analysis, interpretation of data, and study coordination. ID and LB: acquisition of data, analysis of data, and revising the manuscript. HW: revising manuscript for medical writing. GD: revising the manuscript. JB: statistical analysis and revising the manuscript. SM: study supervision and revising the manuscript. AJ: study concept, revising the manuscript, study supervision, and supervision of statistical analysis. All authors contributed to the article and approved the submitted version.

## Conflict of Interest

HW receives honoraria for acting as a member of Scientific Advisory Boards and as consultant for Biogen, Evgen, MedDay Pharmaceuticals, Merck Serono, Novartis, Roche Pharma AG, Sanofi-Genzyme, as well as speaker honoraria and travel support from Alexion, Biogen, Cognomed, F. Hoffmann-La Roche Ltd., Gemeinnützige Hertie-Stiftung, Merck Serono, Novartis, Roche Pharma AG, Sanofi-Genzyme, TEVA, and WebMD Global. He is acting as a paid consultant for Abbvie, Actelion, Biogen, IGES, Johnson & Johnson, Novartis, Roche, Sanofi-Genzyme, and the Swiss Multiple Sclerosis Society. His research is funded by the German Ministry for Education and Research (BMBF), Deutsche Forschungsgemeinschaft (DFG), Else Kröner Fresenius Foundation, Fresenius Foundation, Hertie Foundation, NRW Ministry of Education and Research, Interdisciplinary Center for Clinical Studies (IZKF) Muenster and RE Children's Foundation, Biogen GmbH, GlaxoSmithKline GmbH, Roche Pharma AG, Sanofi-Genzyme. JK received honoraria for lecturing from Biogen, Novartis, Genzyme, Roche, Merck, Mylan, and Teva and financial research support from Sanofi Genzyme and Novartis. SM receives honoraria for lecturing and travel expenses for attending meetings from Almirall, Amicus Therapeutics Germany, Bayer Health Care, Biogen, Celgene, Diamed, Genzyme, MedDay Pharmaceuticals, Merck Serono, Novartis, Novo Nordisk, ONO Pharma, Roche, Sanofi-Aventis, Chugai Pharma, QuintilesIMS, and Teva. His research is funded by the German Ministry for Education and Research (BMBF), Deutsche Forschungsgesellschaft (DFG), Else Kröner Fresenius Foundation, German Academic Exchange Service, Hertie Foundation, Interdisciplinary Center for Clinical Studies (IZKF) Muenster, German Foundation Neurology and Almirall, Amicus Therapeutics Germany, Biogen, Diamed, Fresenius Medical Care, Genzyme, HERZ Burgdorf, Merck Serono, Novartis, ONO Pharma, Roche, and Teva. AJ received honoraria and travel expenses unrelated to this work from Actelion Pharmaceuticals and Novartis. The remaining authors declare that the research was conducted in the absence of any commercial or financial relationships that could be construed as a potential conflict of interest.
