# Supplementary material for: The Agony of Choice? Preserved Affective Decision Making in Early Multiple Sclerosis
Source: Front Neurol. 2020 Sep 2;11:914. doi: 10.3389/fneur.2020.00914 (PMC7492612; doi:10.3389/fneur.2020.00914)
Supplement: Supplementary file 1 [file Table_1.DOCX]

| Supplementary Table 1. Pearson correlations of IGT outcomes with demographic & clinical and cognitive parameters. | | | | | | | | | |
| --- | --- | --- | --- | --- | --- | --- | --- | --- | --- |
|  | **Age,y** | **Education,y** | **EDSS** | **Disease Duration,y** | **SDMT** | **PASAT 3s** | **HADS Anxiety** | **HADS Depression** | **FSMC** |
| **Total Netscore** | -.083 | .227 | -.258 | -.096 | .336 | .285 | -.083 | -.369 | .031 |
| **Total Money** | -.084 | .297 | -.391 | -.340 | .117 | .058 | -.022 | .234 | .119 |
| **Learning Index** | .075 | -.089 | -.211 | -.388 | .002 | .083 | .115 | .176 | .080 |
| Note. EDSS = Expanded disability status scale; FSMC = Fatigue scale for motor and cognitive functions; HADS = Hospital anxiety and depression scale; IGT = Iowa gambling task; PASAT 3s = Paced auditory serial addition test 3 seconds; SDMT = Symbol digits modalities test; y = years; * = indicates a significant correlation, *p*<0.05 (two-tailed); ** = *p*<0.01 (two-tailed); alpha-levels are uncorrected. | | | | | | | | | |
